# Progress in Electrode Modifiers for Nitrite Electrochemical Sensing Applications

**DOI:** 10.3390/bios15120783

**Published:** 2025-11-27

**Authors:** Mohammad Aslam, Saood Ali, Khaled Hamdy, Danishuddin, Khursheed Ahmad, Rohit Kumar Singh Gautam

**Affiliations:** 1 School of Chemical Engineering, Yeungnam University, Gyeongsan 38541, Republic of Korea; 2 School of Mechanical Engineering, Yeungnam University, Gyeongsan 38541, Republic of Korea; 3 Department of Biotechnology, Yeungnam University, Gyeongsan 38541, Republic of Korea; 4 Discipline of Chemistry, Indian Institute of Technology Indore, Indore 453552, India; 5 Department of Mechanical Engineering, Teerthanker Mahaveer University, Moradabad 244001, India

**Keywords:** nitrite, food safety, environmental pollutants, electrochemical sensors/biosensors

## Abstract

It is well known that nitrite is widely used in industrial and agricultural sectors as a preservative, corrosion inhibitor, and intermediate in chemical synthesis; consequently, nitrite residues are often present in food, water, and the environment as a result of meat curing, fertilizer use, and wastewater discharge. Despite having several applications, nitrite exerts toxic effects on human beings and aquatic life. Therefore, the monitoring of nitrite is of particular significance to avoid negative impacts on human health, the environment, and aquatic life. Previously, the electrochemical method has been extensively used for the development of nitrite sensors using various advanced electrode materials. Additionally, zinc oxide (ZnO), cerium oxide (CeO_2_), titanium dioxide (TiO_2_), copper oxide (CuO), iron oxides, nickel oxide (NiO), polymers, MXenes, reduced graphene oxide (rGO), carbon nanotubes (CNTs), graphitic carbon nitride (gCN), metal–organic frameworks (MOFs), and other composites have been utilized as electrocatalysts for the fabrication of nitrite electrochemical sensors. This review article provides an overview of the construction of nitrite sensors using advanced electrode materials. The electrochemical activities of the reported nitrite sensors are discussed. Furthermore, limitations and future perspectives regarding the determination of nitrite are discussed.

## 1. Introduction

Food safety and a neat and clean environment are two important considerations in today’s world [1,2,3]. The contamination of food and water resources via industrial and agricultural practices has grown as a global concern [4]. Nitrite is one of the environmental pollutants that needs significant attention due to its hazardous impacts on human health and the environment [5,6,7,8]. Despite being an environmental pollutant, nitrite has attracted a great deal of interest because of its significant role in various applications, such as in the food and agriculture industries [9,10,11]. Nitrite is a well-known preservative used in the food industry and for curing fruits, vegetables, fish, and meats [12,13,14,15]. Although nitrite is beneficial for preservation and food safety, its overuse may cause cancer, which is a major concern for human health. It is also understood that harmful bacteria can be inhibited by nitrite; furthermore, it can interact with amines (secondary amines) to form nitrosamines under acidic conditions, which may cause cancer [12]. Long-term consumption of nitrite-containing foods may cause health issues in human beings [16]. Therefore, monitoring of nitrite is necessary for human health, food safety, and a safe environment.

**Scheme 1:**
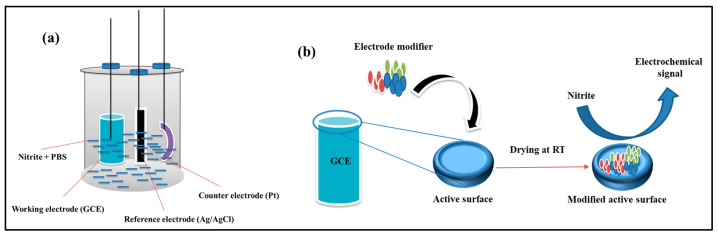
Schematic representation of the three-electrode system (**a**) and electrode modification process (**b**) for nitrite detection.

Conventional methods such as colorimetry [17], chromatography [18], chemiluminescence [19], and capillary electrophoresis [20] can be used for the monitoring of nitrite due to their decent sensitivity. However, such conventional methods have their limitations, such as high cost, a complicated working process, and a long reaction time [21,22]. Thus, it is necessary to develop or explore alternative low-cost approaches for the monitoring of nitrite [23,24,25,26]. Electrochemical methods have attracted a great deal of interest and attention from researchers due to their excellent sensitivity, good selectivity in the presence of interfering species, reproducibility, reasonable recovery in real samples, and stability [27,28,29,30]. The electrochemical sensing method involves a three-electrode system, as displayed in Scheme 1a. The working electrode (WE) consists of a glassy carbon electrode (GCE), whereas platinum (Pt) and silver/silver chloride (Ag/AgCl) electrodes are used as counter and reference electrodes, respectively. It has been found that bare GCE exhibits poor catalytic behavior; therefore, it is necessary to perform surface modification of GCE by employing catalytic materials as electrode modifiers. In this regard, various catalytic materials, such as tungsten sulfide [31], copper bromide [32], gold nanoparticles (Au NPs) [33], cerium (III)-doped copper NPs [34], platinum (Pt) NPs [35], nickel–manganese spinel oxide [36], tungsten oxide (WO_3_) [37], and other materials [38,39,40], have been explored as sensing materials for nitrite detection. Electrode modification with catalytic materials is displayed in Scheme 1b. Electrochemical techniques, including differential pulse voltammetry (DPV), linear sweep voltammetry (LSV), chronoamperometry (CA), cyclic voltammetry (CV), square wave voltammetry (SWV), and amperometry (Amp), can be used for the monitoring of nitrite.

This review article provides an overview of the reported electrode materials for the construction of nitrite electrochemical sensors. The current limitations and future directions for nitrite detection are also discussed. It is believed that this review report may update researchers on recent progress in the development of electrode modifiers towards the detection of nitrite using electrochemical methods.

## 2. Progress in Nitrite Detection

### 2.1. Zinc Oxide (ZnO)-Based Materials

**Scheme 2:**
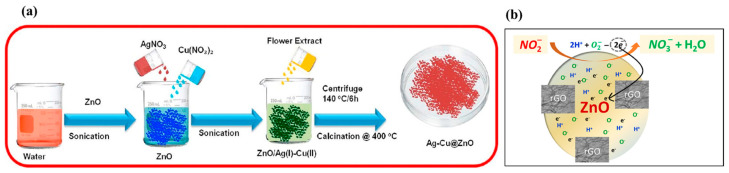
(**a**) This schematic graph shows the formation of Ag-Cu@ZnO. Reproduced with permission [41]. (**b**) Schematic illustration of the reaction mechanism for nitrite sensing. Reproduced with permission [42].

ZnO is one of the semiconducting metal oxides that display excellent catalytic properties for electrochemical applications. It is also understood that functionalization or doping strategies may further enhance the conductivity and electrocatalytic behavior of ZnO and its composite-based electrode materials. Thus, Manjari et al. [41] proposed the incorporation of silver–copper (Ag-Cu) to ZnO, as demonstrated in Scheme 2. It can be seen that silver nitrate and copper nitrate were used as Ag and Cu sources, respectively. The authors used *Acacia caesia* flower extract as the reducing agent for the formation of Ag-Cu-NP-decorated ZnO. The synthesized Ag-Cu@ZnO was adopted as an efficient electrode material and deposited on the GCE surface, which was explored for the electrochemical detection of nitrite using CV and LSV techniques. The performance of the sensors was evaluated in terms of sensitivity, limit of detection (LOD), and linear range. The Ag-Cu@ZnO/GCE displayed an LOD of 17 μM with decent selectivity in the presence of interfering species, such as glucose, copper chloride, sodium chloride, urea, and magnesium chloride. This work exhibited several advantages, such as green synthesis and scalability, for the preparation of Ag-Cu@ZnO towards the construction of nitrite sensors. However, it can be understood that incorporation of the conducting materials, including reduced graphene oxide (rGO) to the ZnO, may enhance the electron transfer process and charge transfer kinetics of the modified electrode. Therefore, rGO/ZnO composite was synthesized via the sol–gel method and the GCE surface was modified with the prepared rGO/ZnO [42]. The rGO/ZnO-modified GCE was adopted as a nitrite electrochemical sensor and its electrochemical capability for the detection of nitrite was checked by using LSV and amperometry techniques.

The LSV technique indicated that electro-catalytic peak current response of the rGO/ZnO/GCE linearly increases with the increase in the nitrite concentration. The GO/ZnO/GCE displayed an LOD of 1.18 µM and 1.36 µM with linear ranges of 200 to 4000 µM and 20 to 520 µM for nitrite detection, using LSV and amperometry methods, respectively. Additionally, rGO/ZnO/GCE also displayed sensitivity of 0.3156 and 0.2754 μA µM^−1^ cm^−2^ using LSV and amperometry, respectively. The rGO/ZnO/GCE exhibited good stability, reproducibility, and selectivity for the detection of nitrite. The improved electrochemical performance may be attributed to the presence of synergism such as high electro-catalytic activity of ZnO and conductivity of rGO in the fabricated rGO/ZnO/GCE. The sensing mechanism for nitrite detection has been depicted in Scheme 2b. The determination of nitrite may involve the formation of oxygen anions and the release of two electrons through the oxidation of nitrite. The oxygen molecules may be adsorbed on catalyst surface as shown in the equations given below.

O_2_ (dissociation) = O_2_ (adsorbed) (1)

O_2_ (adsorbed) + 2e^−^ = 2O_2_^−^ (adsorbed) (2)

NO_2_^−^ +2 H^+^ + O_2_^−^ (adsorbed) = NO_3_^−^ + H_2_O + 2e^−^ (3)

**Figure 1:**
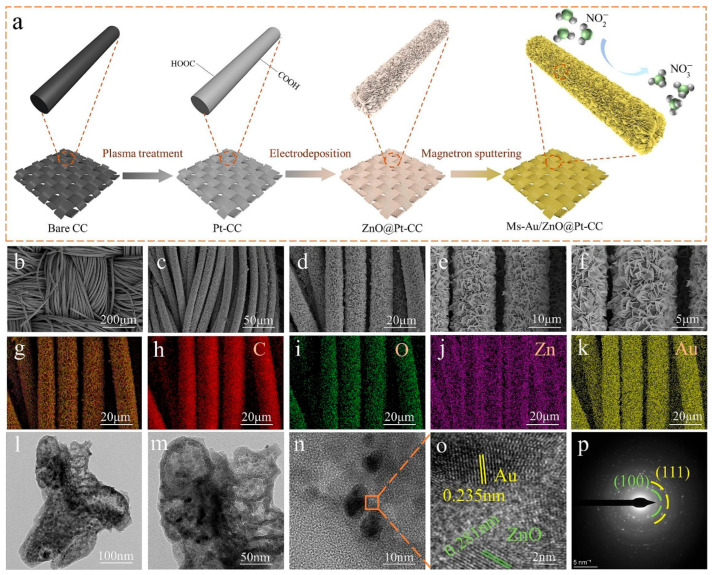
(**a**) A schematic graph showing the preparation of Ms-Au/ZnO@Pt/CC. SEM images (**b**–**f**) of Ms-Au/ZnO@Pt/CC. EDX mapping image (**g**–**k**) of Ms-Au/ZnO@Pt/CC. TEM images (**l**,**m**) of Ms-Au/ZnO@Pt/CC and HR-TEM images (**n**,**o**) of Ms-Au/ZnO@Pt/CC. SAED picture (**p**) of Ms-Au/ZnO@Pt/CC. Reproduced with permission [49].

The surface topology and structural properties of the catalyst may influence the sensitivity of the electrochemical sensors. In this context, Cheng et al. [43] also reported the preparation of the stir-bar-shaped ZnO nanorods (NRs). It was found that prepared ZnO NRs has width of 40 to 90 nm and length of 50 to 170. The stir-bar (sb)-shaped ZnO NRs exhibited reasonable electro-catalytic activity for nitrite detection and the constructed electrode (ZnO/nafion/GCE) displayed a wide linear range of 0.3 μM to 6.16 mM (via amperometry) and 0.8 μM to 4.56 mM (via LSV) with LOD of 0.21 μM (via amperometry) and 0.62 μM (via LSV) for the determination of nitrite. It can be expected that presence of sb-shaped NRs of ZnO facilitated the electron transfer and charger transfer kinetics of the modified electrode for nitrite detection. In addition, ZnO/nafion/GCE showed good stability, sensitivity, and reproducibility for nitrite detection. The practical ability of the ZnO/nafion/GCE was examined in pickles, ham sausage and tap water samples which delivered acceptable nitrite recovery. Although the above-mentioned nitrite sensor displayed promising sensing performance for nitrite detection, the presence of nafion has limitations. The nafion may decrease the electro-catalytic activity of the proposed nitrite sensor. In future studies, this point can be considered. Cheng et al. [44] also proposed incorporating stir bar-shaped ZnO with nitrogen-doped rGO (sb-ZnO/N-rGO) through the hydrothermal method. The obtained sb-ZnO/N-rGO was coated on the GCE surface using facile conditions. The fabricated sb-ZnO/N-rGO/GCE exhibited excellent electro-catalytic activity towards nitrite detection. The presence of the synergistic interactions between the sb-ZnO and N-rGO enhanced the sensitivity of sb-ZnO/N-rGO/GCE for nitrite detection. The fabricated nitrite electrochemical sensor demonstrated an LOD of 0.13 μM with two wide linear ranges of 0.2 to 1200 μM and 1500 to 7800 μM. The sb-ZnO/N-rGO/GCE was also selective for nitrite detection in the presence of interfering species. In addition, sb-ZnO/N-rGO/GCE displayed reasonable recovery of nitrite in tap water, ham sausage, and pickled samples. This proposed nitrite sensor has promising characteristics for practical applications. In another study, Cheng et al. [45] also explored the electrochemical sensing behavior of the spherical-shaped ZnO for nitrite detection. The ZnO was synthesized using the solvothermal method and obtained ZnO was deposited on GCE surface using facile conditions. The electrochemical performance of the fabricated ZnO-based electrode was studied by using chronoamperometry (CA) and LSV techniques. The authors found that the proposed nitrite sensor exhibited two linear ranges of 0.6 to 220 μM and 460 to 5500 μM using the CA method. In contrast, linear ranges of 1.9 to 800 μM and 1080 to 5900 μM were obtained for nitrite detection using the LSV technique. Sensitivity of 0.785 μA μM^−1^ cm^−2^ and 0.646 μA μM^−1^ cm^−2^ with LOD of 0.39 μM and 0.89 μM was obtained for nitrite detection using CA and LSV techniques, respectively. In addition, real sample studies focused on a pickle sample revealed its promising role, which may make it suitable for commercialization. Chowdhury et al. [46] have prepared a novel polypyrrole/titanium oxide (PPy/TiO_2_)/ZnO composite through a chemical oxidative polymerization approach. The PPy/TiO_2_/ZnO-modified electrode exhibited an LOD of 0.14 µM, sensitivity of 70.3234 μA μM^−1^ cm^−2^, and a linear range of 1 to 20 μM. Although this study reported an excellent sensitivity value, the authors focused on multiple applications. Therefore, reproducibility may be compromised in such scenarios. Another study [47] reported the synthesis of a novel hybrid composite of polyaniline (PA)/tetra-amino cobalt phthalocyanine-functionalized ZnO. The surface of the GCE was modified with the synthesized samples. The ZnO, TaCoPc@ZnO, PA-TaCoPc, and PA-TaCoPc@ZnO-modified GCE were used as electrochemical sensors for nitrite detection. It was found that PA-TaCoPc@ZnO/GCE exhibited improved electro-catalytic activity for nitrite detection. The hierarchical near-spherical ZnO (ns-ZnO)/N-rGO composite was also synthesized via the solvothermal method [48]. The ZnO (ns-ZnO)/N-rGO was adopted as sensing layer to modify the surface of the GCE for non-enzymatic detection of nitrite. The hierarchical design may have enhanced the surface area and active sites which facilitated the electron transfer mechanism. Therefore, the fabricated nitrite sensor demonstrated interesting LOD of 0.08 μM with a wide linear range of 0.037 to 5900 μM. The Au NPs are conducting and catalytic materials which can be used to provide conductive support, or as a catalyst or co-catalyst for various electrochemical applications. Therefore, Duan et al. [49] explored the fabrication of Au NPs-decorated ZnO nanoflake arrays on carbon cloth (CC) via the magnetic sputtering (Ms) method. The synthesis of Ms-Au/ZnO@Pt/CC is shown in Figure 1a. The fabricated Au/ZnO@Pt/CC was analyzed by the X-ray diffraction (XRD) method, which confirmed the formation of Au/ZnO@Pt on CC surface. Scanning electron microscopic (SEM) images of the Au/ZnO@Pt/CC at different magnifications are shown in Figure 1b–f. It can be seen that Au/ZnO nanoflowers arrays are wrapped around the carbon fibers’ surface. There was no significant aggregation observed for Au NPs. The energy-dispersive X-ray spectroscopic (EDX) mapping images for the prepared Au/ZnO@Pt/CC are shown in Figure 1g–k, which indicates the uniform elemental distribution. The transmission electron microscopic (TEM) and high-resolution (HR) TEM images of the Au/ZnO@Pt/CC are presented in Figure 1l–o, respectively. The selected area electron diffraction (SAED) image for the Au/ZnO@Pt/CC is shown in Figure 1p. The fabricated Ms-Au/ZnO@Pt/CC was explored for nitrite detection and exhibited a wide linear range of 0.2 to 4986 μM with an LOD of 0.09 μM and high sensitivity of 5677 μA mM^−1^ cm^−2^ towards the determination of nitrite.

Song et al. [50] prepared three-dimensional (3D) cross-linked ZnO (cl-ZnO) nanosheets (NSs) by using the one-pot hydrothermal method for the construction of nitrite sensors. The EIS studies indicated that the fabricated electrode had decent conductivity; subsequently, nitrite detection studies were performed using CA and LSV techniques. The CA and LSV techniques revealed wide linear ranges (CA: 0.8 μM to 462 μM and 0.608 to 7840 μM; LSV: 0.95 to 515 μM and 0.667 to 5410 μM), a low LOD (CA: 0.26 μM; LSV: 0.32 μM), high sensitivities (CA: 824.6 μA·mM^−1^·cm^−2^; LSV: 1336.1 μA·mM^−1^·cm^−2^), and decent selectivity for nitrite detection. The 3D ZnO nanoflowers (ZnO-nf) composed of NRs were also prepared via the hydrothermal method [51]. The ZnO-nf/Nafion-modified GCE demonstrated excellent performance for the detection of nitrite in real samples. This suggests that ZnO-based materials are promising electrode materials for the monitoring of nitrite in food samples. The electrochemical parameters of the above-discussed electrochemical sensors are displayed in Table 1.

### 2.2. Co_3_O_4_-Based Materials

Cobalt oxide (Co_3_O_4_) has excellent electro-catalytic properties for electrochemical reactions. The electrochemical properties of the Co_3_O_4_ may be further improved via a suitable doping strategy. Therefore, N-doped Co_3_O_4_ functionalized with CH_6_N_3_^+^, NH_3_^+^ groups was prepared through the hydrothermal method [52]. L-Arginine was also incorporated to fabricate the nitrite electrochemical sensor, which displayed decent sensitivity and electrochemical performance for nitrite detection. The improved performance may be attributed to the facilitated electron transfer (due to the N-doping and surface functional groups) and improved active sites or area from additional Co^0^/Co^2+^ sites. Zhe et al. [53] incorporated Co_3_O_4_ nanoflowers with ultrafine molybdenum oxide (MoO_3_) NPs to form the (MoO_3_/Co_3_O_4_ composite using a hybrid electrochemical deposition approach. The obtained MoO_3_/Co_3_O_4_ on the CC electrode demonstrates enhanced electro-catalytic activity for nitrite oxidation, which can be ascribed to the synergistic interactions within the MoO_3_ and Co_3_O_4_ heterostructure. The fabricated electrode showed a rapid response time of 2 s, high sensitivity of 1704.1 μA mM^−1^ cm^−2^, and an LOD of 0.075 μM. It was also found that the proposed sensor can be used for the detection of nitrite in water and sausage samples. This suggests that it has potential for practical and commercial applications. The Co_3_O_4_ nanoflakes were also grown on iron (Fe) foam (Co_3_O_4_/Fe) by using the hydrothermal method followed by thermal calcination [54]. The Co_3_O_4_/Fe foam exhibits an improved surface area and catalytic behavior for nitrite detection, which displayed an LOD of 18.4 μM and wide linear range of 2 to 1610 μM with high sensitivity of 3.451 mA mM^−1^ cm^−2^. These reports showed that Co_3_O_4_-based composites have promising properties for the construction of a nitrite electrochemical sensor.

### 2.3. CeO_2_-Based Materials

**Figure 2:**
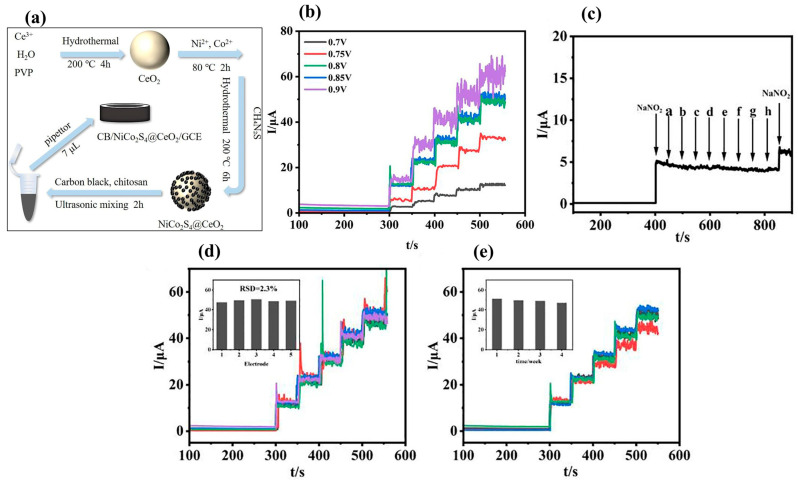
(**a**) A schematic graph representing the formation of a CB/NiCo_2_S_4_@CeO_2_-based electrochemical sensor. (**b**) Amperometric curves of the CB/NiCo_2_S_4_@CeO_2_-modified GCE for nitrite detection at different applied potential values. (**c**) Selectivity (interferences; a = NaCl, b = MgSO_4_, c = KBr, d = NaAc, e = glucose, f = CaCl_2_, g = Na_2_CO_3_, h = trisodium citrate dihydrate), (**d**) reproducibility and (**e**) stability results for nitrite detection. Reproduced with permission [56].

Cerium oxide (CeO_2_) is one of the promising metal oxides used for the electrochemical detection of nitrite. This doping strategy may also improve the electrochemical performance of the CeO_2_. Thus, tin (Sn)-doped CeO_2_ was prepared using the precipitation method for the determination of nitrite [55]. The Sn-CeO_2_-modified GCE exhibited an enhanced electrochemical performance due to its improved surface properties and fine-grained morphology, which facilitated the electron transfer. This sensor also demonstrated an LOD of 16 nM and satisfactory nitrite recovery in environmental water samples. Additionally, the Sn-CeO_2_/GCE also displayed good reproducibility, long-term stability, and selectivity, which highlights its potential as a simple, sensitive, and reliable platform for nitrite sensing. The NiCo_2_S_4_@CeO_2_ composite was also combined with carbon black (CB) to develop the CB/NiCo_2_S_4_@CeO_2_-based nitrite sensor [56]. The fabrication of the electrochemical sensor is illustrated in Figure 2a. The amperometric results for nitrite detection at different applied voltages are displayed in Figure 2b. It was found that an applied voltage of 0.8 V elucidated a promising response for nitrite detection, and thus this was selected as the optimized voltage for further studies. The CB/NiCo_2_S_4_@CeO_2_/GCE exhibited a wide linear range of 0.2 to 7400 μM, sensitivity of 470 μA mM^−1^ cm^−2^, and an LOD of 0.003 μM for nitrite detection via the amperometry method. The CB/NiCo_2_S_4_@CeO_2_/GCE also displayed good selectivity (Figure 2c) and reproducibility (Figure 2d) for nitrite detection. The proposed nitrite sensor exhibited good stability lasting 30 days (Figure 2e). These results suggest that synthesized CB/NiCo_2_S_4_@CeO_2_ is a robust and promising material for sensitive nitrite detection.

The CeO_2_-based electrode materials (CeO_2_/rGO; Cu@CeO_2_/rGO) were prepared using the electrochemical deposition approach [57]. The CeO_2_/rGO was directly coated on pencil graphite electrodes (PGEs) and its surface was further enhanced with Cu NPs to improve the sensing of nitrite. The Cu@CeO_2_/rGO electrode demonstrated an LOD of 10.1 nM, high sensitivity of 1963.2 μA μM^−1^ cm^−2^, and a wide dynamic linear range of 10 to 2000 μM with excellent selectivity for nitrite detection. Zhang et al. [58] also reported a novel p-CuO nanoflowers/n-CeO_2_ NSs heterojunction anchored on CC for the determination of the nitrite sensor. It was also observed that p-CuO nanoflowers/n-CeO_2_ NSs show improved surface areas and active sites, which may facilitate the electron transfer. The formation of p-n heterojunction may improve carrier mobility, Ce^3+^ and oxygen vacancy concentration, and electron transfer efficiency, which may further boost the electro-catalytic activity of the fabricated electrode for nitrite detection. The CuO NFs/CeO_2_ NSs/CC sensor exhibited a wide linear range of 0.1 to 4000 μM, high sensitivity of 11,610 μA mM^−1^ cm^−2^, and a low LOD of 0.037 μM. The proposed sensor also demonstrated excellent selectivity, reproducibility, repeatability, and stability. The acceptable recovery of nitrite in real samples of items such as Coca Cola, pickled cabbage, and drinking water suggested its potential for commercialization.

### 2.4. Iron Oxide-Based Materials

**Figure 3:**
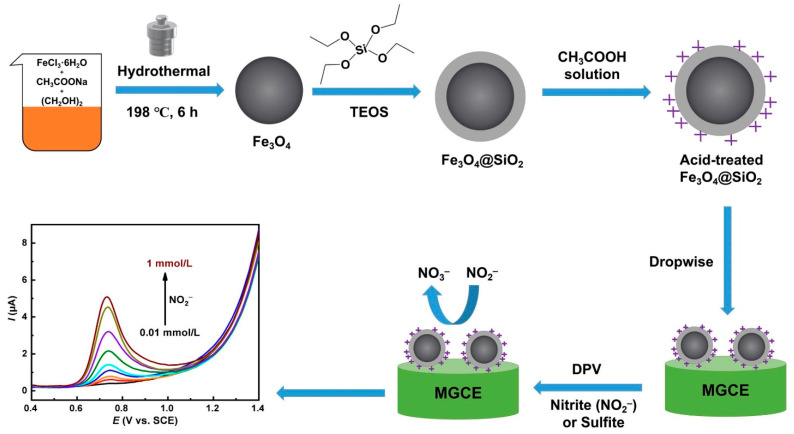
Schematic graphs of the preparation of Fe_3_O_4_@SiO_2_/MGCE for electrochemical sensing applications. Reproduced with permission [62].

Riahifar et al. [59] employed a novel electrochemical sensor for the determination of nitrite using cysteine-functionalized Fe_3_O_4_@Au core–shell nanoparticles (Fe_3_O_4_@Au@Cys)/rGO as electrode modifiers. The constructed Fe_3_O_4_@Au@Cys/rGO/GCE displayed improved electro-catalytic activity for the monitoring of nitrite using voltammetric techniques. The Fe_3_O_4_@Au@Cys/rGO/GCE demonstrated a decent reproducible response with two wide linear detection ranges of 0.03 to 344 µM and 344 to 2215 µM, along with a low LOD of 8 nM. The real samples evaluated for nitrite detection were also examined in human serum, urine, and water samples, which displayed satisfactory recovery values. Song et al. [60] also prepared NiO/Fe_2_O_3_ and coated it on a GCE surface for the determination of nitrite. The observations showed that NiO/Fe_2_O_3_-modified GCEs exhibit an LOD of 0.05 μM with a wide linear range of 5 to 500 μM for nitrite detection. The synergism in the prepared electrode material facilitated the electron transfer and enhanced the sensitivity of nitrite detection. Furthermore, it was also found that Fe_2_O_3_/Fe_3_C/Fe NPs-anchored carbon spheres (CSs@Fe_2_O_3_/Fe_3_C/Fe) have the potential to determine the nitrite content in food samples [61]. The synergistic combination of carbon sphere-mediated adsorption and the electro-catalytic activity of the composite enhance the determination of nitrite at the surface of modified GCEs. High sensitivity of 451.85 µA cm^−2^ mM^−1^, a low LOD of 0.06 µM, and a wide linear range of 1 to 2540 µM, with good selectivity and stability, were also observed under the optimized conditions. The acid-treated Fe_3_O_4_@SiO_2_ NPs were also explored for the detection of nitrite and sulfite [62]. The materials’ preparation and the fabrication of the electrode are depicted in Figure 3.

Briefly, Fe_3_O_4_@SiO_2_ NPs were synthesized by employing sol–gel and hydrothermal methods. The synthesized material exhibited positive surface charges after acid treatment and enhanced electrostatic interactions with nitrite and sulfite. The Fe_3_O_4_@SiO_2_ NPs-modified electrode exhibited an LOD of 3.33 μM for nitrite with excellent selectivity, reproducibility, and repeatability. In another study [63], a metal–organic framework (MOF) was combined with Fe_3_O_4_@Au to form the Fe_3_O_4_@Au/MOF using a green self-assembly approach where waste orange peel acted as a reducing agent. The vanadium-substituted tungsten phosphate (K_7_P_2_W_17_VO_62_·18H_2_O, P_2_W_17_V) was also combined with Fe_3_O_4_@Au/MOF and deposited onto the GCE surface via layer-by-layer self-assembly and electro-deposition approaches. The constructed electrode (Fe_3_O_4_@Au/MOF-P_2_W_17_V) exhibited a wide linear range of 0.01 to 100 mM, high sensitivity of 11.682 μA·μM^−1^·cm^−2^, and a low LOD of 0.532 μM for nitrite detection. In another study [64], LSV technique-based electrochemical studies revealed that nitrite can be detected with an LOD of 2.25 mM, sensitivity of 73.84 µA·mM^−1^·cm^−2^, and a linear range of 0.74 to 1.09 M using GO@Fe_2_O_3_/Y_2_O_3_ as the electrode material. The electrochemical performance of the Co_3_O_4_, CeO_2_ and iron oxide-based materials for nitrite detection have been summarized in Table 2.

### 2.5. CuO-Based Materials

**Figure 4:**
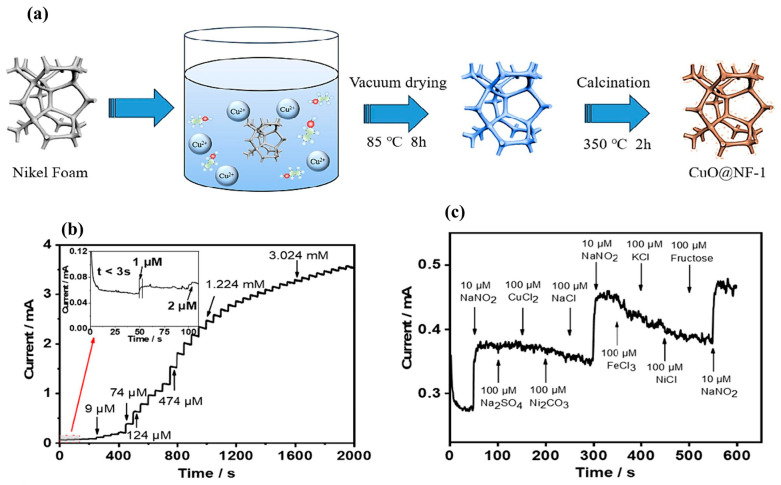
(**a**) Schematic graph for CuO@NF-1 preparation. Amperometry curves for different concentrations (**b**) and selectivity test (**c**). Reproduced with permission [67].

Copper oxide (CuO) is well-known electrode material for the construction of electrochemical sensors for the monitoring of environmental pollutants. The electrochemical performance of the CuO can be further improved by incorporating it with other materials. Therefore, the CuO/NiO composite was fabricated on FTO substrate using facile conditions [65]. It was found that leaf-like CuO improved the active area of the electrode and lowered the charge transfer resistance, whereas NiO facilitated the electron transfer and minimized the diffusion resistance. Thus, the fabricated electrode demonstrated an LOD of 0.013 μM and a linear range of 0.001 to 1.8 mM with excellent stability. In another study [66], CuO NPs/CC were fabricated and their electrochemical activity for nitrite detection was determined using the electrochemical method. The wide linear range of 0.5 to 3000 μM, LOD of 0.043 μM, and high sensitivity of 1656 μA mM^−1^ cm^−2^ were used for nitrite detection. Furthermore, the CuO NPs/CC electrode demonstrated reliable nitrite determination in real samples, including samples of drinking water and sauerkraut, with satisfactory recovery rates, suggesting that it has potential for practical applications. The CuO nanocatalyst self-supported nickel foam (CuO@NF) was synthesized through a simple vacuum-assisted solvent method followed by thermal annealing as shown in Figure 4a [67].

It was observed that a sea urchin-like CuO structure was formed with 3D porous architecture and it displayed a high surface area which may promote nitrite detection. The proposed electrochemical sensor exhibited wide linear range of 0.5 to 4250 μM with LOD of 0.5 μM for nitrite detection. The current response increases with the addition of nitrite as shown in Figure 4b. The proposed sensor also exhibited decent selectivity for nitrite detection (Figure 4c). The proposed electrode also demonstrated reliable nitrite detection in real samples such as pickled vegetables. This work highlighted strategic approaches for the fabrication of a binder-free nitrite electrochemical sensor.

### 2.6. TiO_2_-Based Materials

Titanium dioxide (TiO_2_) is a semiconducting metal oxide which can be used for the development of electrochemical sensors. The low conductivity of TiO_2_ is the most notable concern with regard to this material; therefore it will be of great significance to incorporate conductive materials into TiO_2_. In this regard, titanium carbide (Ti_3_C_2_T_x_) MXene was incorporated with TiO_2_ to fabricate the nitrite sensor [68]. This fabricated novel nitrite sensor displays good recovery of nitrite in milk samples and water samples, suggesting that it has potential for practical applications in food samples. In another study [69], 3D mesoporous NRs (MNRs) of peeled montmorillonite (MMT)/TiO_2_/ZnO composite were prepared and its electrochemical activity for nitrite detection was assessed using SWASV. The fabricated sensor exhibited a wide linear response of 0.04 to 10 nM, a low LOD of 0.12 nM, and sensitivity of 0.78 μA nM^−1^, with excellent selectivity. Raghu et al. [70] explored the use of Au-doped TiO_2_ NPs as a sensing material for the determination of nitrite using the SWV technique. This nitrite sensor was capable of detecting nitrite with an LOD of 0.095 µM and a wide dynamic linear range of 3.3 to 120 µM. The real sample analysis with acceptable recovery and long-term stability suggested its potential for practical applications. The CPE modified with PANI-TiO_2_/Pt composites was explored for the construction of nitrite sensor, which demonstrated a decent electrochemical performance [71].

### 2.7. MoS_2_-Based Materials

**Figure 5:**
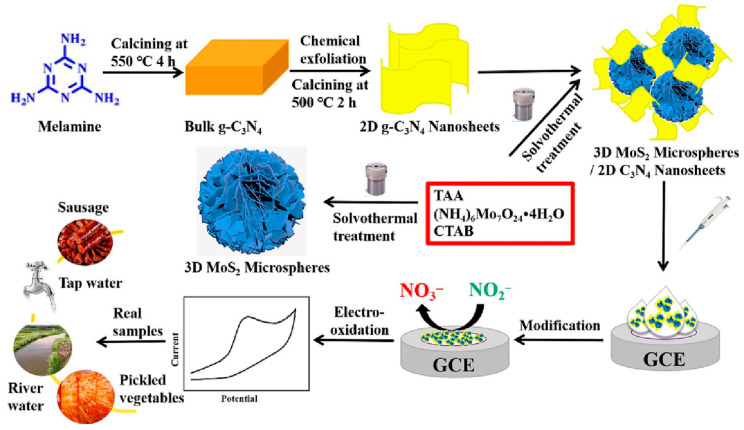
Schematic graphs for the fabrication of MoS_2_/gCN/GCE. Reproduced with permission [77].

It is well understood that molybdenum disulfide (MoS_2_) possesses good electro-catalytic properties for electrochemical reactions. The Ni/MoS_2_ was synthesized for the fabrication of nitrite electrochemical sensors [72]. The Ni/MoS_2_ electrode exhibited reliable nitrite sensing with a linear range of 5 to 800 μM and a low LOD of 2.48 μM. This suggests that it has potential for nitrite detection in food samples. Another study also reported oxidized MoS_2_ as sensing material for electrochemical detection of nitrite; it exhibited a linear range of 1 μM to 386 μM with an LOD of 0.028 μM [73]. These studies explained the role of MoS_2_ oxidation in enhancing electrochemical activity and displayed the potential of layered nanostructures for environmental and food safety monitoring applications. Zhang et al. [74] prepared a petal-shaped FeS@MoS_2_/C composite through the hydrothermal method. This prepared composite was combined with hemoglobin (Hb). The nafion was drop-casted onto a carbon ionic liquid electrode (CILE) to fabricate the nitrite sensor. The fabricated nafion/Hb/FeS@MoS_2_/C/CILE displayed good recovery of nitrite in pickle juice samples. A novel composite (AuNPs@MoS_2_/rGO) was obtained using hydrothermal and chemical reduction approaches [75]. The electrochemical performance of the AuNPs@MoS_2_/rGO was tested for nitrite detection and exhibited excellent sensitivity of 0.805 μA·μM^−1^·cm^−2^ and a low LOD of 0.038 μM with a wide linear range of 0.2 to 2600 μM. The enhanced performance was attributed to the synergistic interaction between MoS_2_ and rGO NSs. The GO/PEDOT:PSS-modified GCE also exhibited decent sensitivity for nitrite detection in terms of a linear range of 1 to 200 μM and an LOD of 0.5 μM [76]. Furthermore, it showed excellent stability, reproducibility, and selectivity for the determination of nitrite. These investigations showed the potential role of GO/PEDOT:PSS-modified GCE as a promising electrochemical sensor for nitrite detection in environmental monitoring and food samples. The flower-like MoS_2_ was combined with graphitic carbon nitride (gCN) to fabricate the MoS_2_/gCN composite [77]. The schematic graph shows the fabrication of MoS_2_/gCN/GCE for nitrite detection (Figure 5).

The MoS_2_/gCN composite leverages synergistic interactions, active sites, a mesoporous 3D structure, and decent conductivity, which enhanced the detection of nitrite. The MoS_2_/gCN composite-modified GCE exhibited a wide linear range of 0.1 to 1100 μM, an LOD of 0.065 μM, and excellent stability, reproducibility, and selectivity. The practical applicability was also confirmed through the successful detection of nitrite in food (sausage and pickled vegetables) and environmental (river and tap water) samples, suggesting that it has potential for real-time monitoring of nitrite in practical applications. Zhang et al. [78] prepared a NiS_x_@MoS_2_ composite using a benign approach for the development of a nitrite electrochemical sensor. This sensor demonstrates a broad detection linear range of 0.0001 to 0.0020 mg mL^−1^ with an LOD of 1.863 × 10^−5^ mg mL^−1^ and excellent selectivity in the presence of various interfering species. These investigations suggest that it holds significant promise for applications in food-safety monitoring and related analytical fields. The electrochemical activities of the CuO-, TiO_2_-, and MoS_2_-based materials for nitrite detection are summarized in Table 3.

### 2.8. Polymer-Based Materials

Faisal et al. [79] reported the fabrication of Au-modified polypyrrole-C/gCN composite (Au@PPy-C/gCN through pyrolysis and an ultrasonication method. The Au@PPy-C/gCN was deposited on the surface of the GCE to develop an electrochemical sensor for nitrite detection. The authors also observed that the proposed electrode material is a promising material for nitrite-sensing applications, as it displayed high sensitivity of 91.19 µA µM^−1^ cm^−2^ and a reasonable LOD of 1.11 µM when the DPV method was used. Wahyuni et al. [80] integrated Au NRs with either electrochemically reduced graphene oxide (ErGO) or MWCNTs combined with PEDOT:PSS for the sensing of nitrite. The constructed AuNRs/ErGO/PEDOT:PSS/GCE and AuNRs/MWCNTs/PEDOT:PSS/GCE were used as electrochemical sensors for the determination of nitrite. The enhanced electro-catalytic activity arose as a result of the synergistic interactions between AuNRs and their composites. It was also found that the MWCNTs-based nitrite sensor displayed a good electrochemical performance due to its high surface area and conductivity, larger electrochemically active surface area (0.1510 cm^2^), and higher diffusion coefficient. Studies based on detecting nitrate in beef samples also displayed satisfactory results. Abbas et al. [81] explored poly(3,4-ethylenedioxythiophene)-functionalized carbon-supported Cu NPs (PEDOT-C@Cu-NPs) through the green synthesis method. Machine learning (ML) technology was employed to optimize key experimental parameters such as pH, drying time, and precursor concentrations to improve the performance of the proposed nitrite sensor. The ML-guided C@Cu-NPs were subsequently functionalized with PEDOT, which was serving as a π-electron mediator. The prepared PEDOT-C@Cu-NPs electrode exhibited a superior electro-oxidation performance with regard to nitrite detection, which was attributed to exposed Cu facets, defect-rich graphitic carbon, and high π-electron density. This sensor demonstrated an LOD of 3.91 μM, high sensitivity of 0.6372 μA μM^−1^ cm^−2^, and a broad linear range of 5 to 580 μM. Moreover, the electrode achieved a robust performance in real-sample analysis, effectively detecting the presence of nitrite in pickled vegetable extracts. Guan et al. [82] also used an Fe_3_O_4_@Au-Cu/MOF nanozyme as a sensor for the determination of nitrite, and it displayed a decent electrochemical performance. The AuNPs@PPy/rGO/GCE was fabricated for the determination of nitrite [83]. These reports showed that polymers or polymer-based composites can be used for the determination of nitrite in water and food samples.

### 2.9. MOF/ZIF-Based Materials

A novel electrochemical sensor was developed for nitrite detection using cobalt and nitrogen co-doped carbon polyhedrons (Co, N-CP) derived from core–shell ZIF-8@ZIF-67 and electrodeposited Ni NPs [84]. Typically, Co, N-CP was synthesized via direct pyrolysis of ZIF-8@ZIF-67 at 900 °C, whereas Ni NPs were then electrodeposited onto a Co, N-CP. Therefore, Co, N-CP/GCE was developed, displaying a linear range of 5 to 1000 μM and an LOD of 0.094 μM. The 2D nickel phthalocyanine-based MOFs (NiPc-MOFs) with high conductivity were also synthesized through the solvothermal method [85]. The large surface area and superior conductivity of the proposed material improved the electro-catalytic properties for nitrite detection, which displayed a wide linear range of 0.01 mM to 11,500 mM and LOD of 2.3 μM. In another study [86], a series of Co- and N-doped porous carbon rods (CoN-PCRs) were also synthesized through the calcination of rod-shaped mixed-metal ZIF-L precursors with varying Co/Zn ratios. The CoN-PCRs-0.6 exhibited the highest surface area and an optimal electrochemical response for nitrite detection and an LOD of 0.14 μM was obtained with two wide linear ranges of 0.2 to 4000 μM and 4000 to 100,000 μM. The sensor also showed excellent selectivity, reproducibility, and stability, enabling rapid and accurate nitrite detection in tap water. Feng et al. [87] also reported the sensing of nitrite using Co-based porous ZIF-67/rGO/NiNPs as an electrode modifier. The ZIF-67C@rGO/NiNPs/GCE exhibited a superior catalytic current response compared to the other fabricated electrodes. The amperometric analysis revealed a decent linear response to nitrite from 0.2 to 123 μM and 123 to 473 μM with an LOD of 0.086 μM. This sensor also showed excellent stability, reproducibility, and selectivity. The Co@N-doped carbon nanorods (CoN-CRs) were synthesized via pyrolysis of a rod-shaped Co-ZIF-L precursor prepared using polyvinylpyrrolidone (PVP) [88]. Compared to the Co@N-doped carbon sheets (CoN-CSs), CoN-CRs showed a superior sensing performance due to their larger surface area and more active sites. The fabricated sensor exhibited two linear ranges of 0.5 to 4000 μM and 4000 to 8000 μM with sensitivities of 1.03 and 0.82 μA μM^−1^ cm^−2^, respectively, and an LOD of 0.17 μM. The sensor also demonstrated strong selectivity, stability, and reproducibility, effectively detecting nitrite in real samples of sausage and tap water. These results highlight CoN-CRs as promising candidates for practical nitrite-monitoring applications. A novel electrochemical sensor was also developed using core–shell ZIF-8@ZIF-67/Au NPs as an electrochemical sensing material [89]. The DPV analysis showed that the proposed sensor can deliver an LOD of 2 nM and a wide linear range of 0.64 × 10^−8^ to 22.14 × 10^−3^ M for nitrite detection. The high sensitivity, excellent selectivity, stability, reproducibility, and repeatability were also key points in this reported study. The SPCE modified with a carbon black/copper metal–organic framework (CB/Cu-MOF) composite was also explored for the determination of nitrite [90]. The CB/Cu-MOF/SPCE sensor demonstrated an LOD of 0.084 µM and a linear dynamic range of 1 to 200 µM. In another study [91], a multiple-step heterophase approach was used to synthesize crystalline–amorphous Zn/Co-Fe porous nanosheets (C-A Zn/Co-Fe PNSs) on CC. The prepared C-A Zn/Co-Fe PNSs@CC interface featured interwoven carbon fibers decorated with uniformly distributed hybrid NSs, creating abundant crystalline–amorphous junctions. The optimized conditions showed a decent LOD of 0.44 μM for nitrite detection with decent sensitivity. Zhang et al. [92] prepared zirconium–copper bimetallic MOF functionalized with the ionic liquid [BMIM][PF_6_] through the wet impregnation method. The ZrCu-MOF-818/IL-modified electrode demonstrated an LOD of 0.148 μM and broad linear ranges of 6 to 3000 μM and 3000 to 5030 μM. This proposed sensor also demonstrated high reproducibility and repeatability. This sensor also displayed acceptable recovery of nitrite in tap water and pickle juice samples. The Cu/Zn-MOF was also prepared for the determination of nitrite and displayed a decent sensing performance [93]. The Ag-based MOF was also explored with regard to the construction of a nitrite electrochemical sensor [94]. The fabricated electrode was deemed efficient for the determination of nitrite in beetroot, spinach, canned chicken, and pond water samples. The octahedral carbon composites embedded with multivalent Cu/CuO_x_ NPs(Cu/CuO_x_@C) were synthesized via carbonization of copper-based MOF [95]. The fabricated Cu/CuO_x_@C electrode demonstrated an LOD of 0.5 μM, high sensitivity of 0.932 μA μM^−1^ cm^−2^, and a wide linear range of 1 to 8000 μM. These studies revealed that MOF-based materials can be used for the determination of nitrite. In another previous study [96], facile room-temperature synthesis of Ni-based MOF (Ni-PDCA) was investigated for the construction of a nitrite electrochemical sensor. It was found that the prepared polyhedral Ni-PDCA crystals provided acceptable conductivity and a larger electro-active surface area which may have enhanced nitrite oxidation. The Ni-PDCA/SPCE showed an LOD of 0.052 µM, high sensitivity of 240 µA mM^−1^ cm^−2^, and a wide linear range of 0.1 to 1000 µM. The ligand nitrite charge transfer further improved the selectivity of the nitrite sensor. The trimetallic zeolitic imidazolate framework (ZnCoMn ZIF-67) was also synthesized, followed by functionalization with DA to form hollow ZnCoMn ZIF-67@DA (H-ZnCoMn ZIF-67@DA) [97]. Furthermore, pyrolysis yielded hollow Co-, Mn-, and N-doped porous carbons (H-CoMnN-PCs) and it was found that the prepared sample retained a hollow morphology. The H-CoMnN-PCs-based nitrite sensor demonstrated two linear ranges of 0.1 to 1500 μM and 1500 to 12,000 μM with an LOD of 0.097 μM along with excellent selectivity, stability, and reproducibility. The electrochemical sensing performances of the polymer-, MOF-, and ZIF-based materials are described in Table 4.

### 2.10. gCN-Based Materials

Graphitic carbon nitride (gCN) is one of the carbon derivatives which possesses excellent physicochemical properties. These properties makes it a promising electrode material for electrochemical sensing applications. In this context, Shi et al. [98] reported the extraction of chitosan from *Ganoderma lucidum* polysaccharides integrated with gCN to fabricate a GCE-based electrode (chitosan/gCN/GCE) for detecting nitrate in water and food samples. Electrochemical studies revealed that the modified electrode exhibited high sensitivity, selectivity, stability, and a wide linear range. The real sample analysis also showed satisfactory recovery, which highlighted the practical applicability of the sensor. The chitosan/gCN composite enhanced electrode performance and showed strong potential for environmental monitoring, food safety, and biomedical diagnostics. Laser-induced fibers (LIFs) emerged as a promising material due to their significant electrochemical activity and benign fabrication. Thus, LIF-functionalized laser-induced graphene (LIG) electrodes were explored for nitrite detection [99]. The LIFs extracted from Kapton through the ultrasonication process were employed to modify LIG surfaces, which increased the active surface area (from 0.669 cm^2^ for bare LIG to 0.83 cm^2^ for LIF/LIG) and enhanced heterogeneous electron transfer rates (from 0.190 to 0.346 cm/s). Further modification of LIF with the copper phthalocyanine (CuPc) composite exhibited synergistic effects (CuPc catalyzed nitrite oxidation whereas LIF facilitated electron transfer). Therefore, an LOD of 0.12 μM, a wide linear range of 10 to 10,000 μM, and high selectivity for nitrite detection were achieved. In another study, an EDAS/gCN-Au composite was synthesized through chemical reduction and employed as a sensing material for electrochemical nitrite detection [100]. The synergistic interaction between Au NPs and gCN enhanced nitrite electro-oxidation. The fabricated sensor demonstrated acceptable sensitivity of 0.0696 µA µM^−1^ cm^−2^, a linear range of 10 to 375 µM, and an interesting LOD of 0.6 µM. This sensor also exhibited good selectivity in the presence of various physiological molecules and major inorganic ions, indicating its potential for real water sample analysis.

### 2.11. CNTs-Based Materials

In the previous investigations, LIG-based flexible electrode was fabricated by using f-MWCNTs/Au NPs as a sensing material [101]. The LIG/f-MWCNT-AuNPs electrode exhibited a superior performance due to the combined effect of MWCNTs and AuNPs, which increased the electro-active surface area and reduced charge-transfer resistance at the electrode–electrolyte interface. The proposed sensor demonstrated excellent reproducibility with a linear range of 10 to 140 μM and an LOD of 0.9 μM. The K(1,1′-(1,4-Butanediyl)dipyridinium)_2_[PW_11_O_39_Co(H_2_O)] was combined with carboxyl-functionalized MWCNTs ((bdpy)PW11Co/MWCNTs-COOH) through the electro-deposition method [102]. The fabricated sensor exhibited a wide linear range of 10 to 1600 µM, an LOD of 0.63 µM, and high sensitivity of 17.9 µA mM^−1^. In addition, the proposed sensor displayed excellent selectivity, repeatability, reproducibility, and reliable recovery in real samples, demonstrating its potential for practical applications. Another study [103] reported the determination of nitrite copper chlorophyllin (CuCP)/MWCNTs composite as a sensing material. The modified electrode exhibited a wide linear range of 10 μM to 10 mM for nitrite detection and the acceptable recovery of nitrite in a human saliva sample suggested its practical applications. The MWCNTs/AuNPs/PM (PM = poly-melamine) were prepared through layer-by-layer self-assembly on the GCE surface [104]. A wide linear range of 0.4 to 1475 μM with an LOD of 0.041 μM was observed for nitrite detection. In another study, a platinum group metal complexes-based nitrite sensor was also developed and exhibited a decent LOD of 0.39 µM and a linear range of 0 to 909 µM using the DPV technique [105]. Shahzad et al. [106] also explored MWCNTs modified with yttrium oxide (Y_2_O_3_) and iron oxide (Fe_2_O_3_) for the development of a nitrite sensor. The CV and LSV techniques were explored for sensing studies and the authors obtained a linear range of 1 to 1.6 M with an LOD of 0.027 M. A novel rotational paper-based device integrating an electrochemical sensor was developed for the simultaneous detection of nitrite and nitrate ions [107]. The SPE modified with N-MWCNTs and copper (II) phthalocyanine (CuPc) displayed enhanced sensitivity for nitrite detection. This proposed sensor exhibited a linear range of 50 to 1000 μM with an LOD of 10 μM for nitrite detection. The successful recovery of nitrite in meat samples also suggested that it has practical applications for food safety. The GCE was also modified with hydroxylated MWCNTs (MWCNTs-OH)/graphene-decorated Au NPs [108]. The synergistic interaction between the AuNPs and the MWCNT-OH/graphene improved the electro-catalytic activity, conductivity, and active sites, which enhanced the detection of nitrite on the electrode surface. The fabricated sensor demonstrated high reproducibility, stability, and selectivity, with satisfactory recoveries of 97 to 112% in tap and lake water. These studies showed that CNTs-based materials displayed good sensing behavior for nitrite detection.

### 2.12. GO and rGO-Based Materials

Electrochemically reduced holey graphene (ERHG) may offer several advantages, including numerous nanopores across its basal plane which generate abundant edge sites and structural defects. Thus, ERHG can enhance electron transfer and facilitate efficient mass transport. An ERHG-modified electrode was used as a nitrite sensor and exhibited a decent electrochemical performance [109]. ERHG exhibited a promising performance, but challenges related to the cost-effectiveness of large-scale synthesis and long-term stability in complex environments need to be addressed before it can be used for practical applications. The GO-PANI-AuNP composite was synthesized through the hydrothermal method and employed as a sensing material for the electrochemical detection of nitrite [110]. It was observed that PANI provides a conductive network while uniformly distributed Au NPs enhance the catalytic properties of the prepared composite. The enhanced active sites and presence of defects on GO surface improved the detection of nitrite with LOD of 0.17 μM and acceptable recovery in tap water and sewage water samples. Commercially available nail polish (NP) was explored to determine its efficiency for the preparation of 3D porous graphene [111]. The fabricated NP-derived LIG (NP-LIG) exhibited high electrical conductivity, mechanical stability, and tunable surface chemistry, making it a well-suited sensing material for electrochemical applications. The chitosan-modified NP-LIG demonstrated a wide linear range of 2 to 1000 μM and an LOD of 0.9 μM. Another study described the fabrication of a laser-based approach to prepare N,O co-doped porous graphene using commercial polyimide (PI) tape [112]. The electrode demonstrated a linear DPV response in the range of 5 to 450 μM and showed an LOD of 0.8 μM. The SPCE was also modified with nickel, poly(diallyldimethylammonium chloride) (PDDA), and rGO [113]. A linear range of 6 to 100 μM, LOD of 1.99 μM and sensitivity of 0.453 μA μM^−1^ cm^−2^ for nitrite detection were achieved under the optimized conditions. The detection of nitrite in sausages and pickled vegetables samples suggested its potential for food safety applications. The ultrathin 2D/2D hematene/GO nanohybrid composite was prepared using facile conditions [114]. The large surface area and active sites of the prepared composite facilitated the electron transfer and enhanced the electrochemical reactions for the sensing of nitrite. The real sample studies were performed in tap, mineral, and river water samples, which displayed satisfactory nitrite recovery. La_0.8_Sr_0.2_MnO_3_ (LSM) microspheres integrated with rGO were also adopted as electrochemical sensing materials [115]. The rGO content was optimized and the 15 wt % rGO-based composite displayed an interesting LOD of 0.016 μM and sensitivity of 0.041 μA μM^−1^ cm^−2^. The one-step electro-deposition approach was also employed to fabricate an Au/NiO/Rh-modified LIG electrode for nitrite-sensing applications [116]. The incorporation of Au and Rh NPs enhanced electron transport, while NiO contributed additional active sites and increased the surface area, collectively producing strong synergistic effects. The fabricated sensor displayed wide linear range of 1 μM^−1^ mM, an LOD of 0.3 μM, and decent selectivity. The LIG electrode was also engineered with Au NPs and glycine-functionalized nanocarbon (Gly-C) for nitrite detection [117]. The incorporation of Au NPs significantly improved the electro-catalytic properties, whereas Gly-C improved the surface hydrophilicity and adsorption capability to improve the detection of nitrite. The fabricated sensor exhibited two linear ranges of 7 to 700 μg/L and 700 to 1050 μg/L with an LOD of 0.84 μg/L. A novel In_2_O_3_-based solution-gated electrochemical transistor (SGET) sensor was also developed for nitrite detection [118]. The PtAu_4_ NPs decorated rGO (PtAu_4_/RGO) composite was electrodeposited on the transistor gate. The sensor demonstrated an interesting LOD of 0.1 nM with a linear range of 0.1 nM to 1 mM. This sensor also maintained decent stability and selectivity for nitrite detection. A solvothermal approach was also adopted to synthesize hybrid nanostructures of rGO integrated with copper sulfide (CuS) microflowers for nitrite-sensing applications [119]. It was observed that rGO has a nanosheet-like surface, whereas CuS consists of microflowers. The proposed novel sensor displayed decent selectivity and sensitivity for nitrite detection. The polyneutral red/rGO paste electrode (pNR/rGO-PE) was also employed as a sensitive and reliable electrochemical sensor for nitrite detection in real food samples [120]. This sensor displayed wide linearity, but its sensitivity for nitrite detection was poor. The non-enzymatic electrochemical nitrite sensor was also developed using methyl red/rGO/CPE for detecting nitrate in food samples [121]. The MR/GO/CPE exhibited sensitivity of 0.12735 μA/μM, a linear range of 0 to 7000 μM, and an LOD of 0.011 μM. The practical applicability of the proposed sensor was also studied in spiked meat samples, which showed acceptable recovery. It is well known that boron doping significantly tunes the electronic properties of carbon materials. In this context, boron-doped porous graphene (B-PG) was synthesized for the detection of nitrite in food samples [122]. The B-PG-modified GCE demonstrated two linear ranges of 3 to 3000 μM and 3000 to 15,000 μM with an LOD of 1.1 μM. This sensor also exhibited high sensitivity, reproducibility, and acceptable recovery in food samples. The CuCo_2_O_4_/rGO composite was obtained through the one-pot electrochemical method. The synthesized material was deposited on PGE for the determination of nitrite [123]. The reported literature shows that graphene-based materials have various advantages such as high conductivity and flexibility for the construction of electrochemical sensors. The sensing performance of the gCN-, CNTs-, GO-, and rGO-based sensors are compiled in Table 5.

### 2.13. MXene-Based Materials

Recently, MXenes have emerged as a promising material for electrochemical sensing applications, owing to their unique combination of metallic conductivity, a large surface area, and tunable surface chemistry. The 2D-layered structure of the MXenes offers abundant active sites for analyte adsorption, whereas the presence of surface terminations (–OH, –O, –F) may enable functionalization for improved selectivity and sensitivity. The high electrical conductivity of the MXenes facilitates electron transfer, making them a desirable material for electrochemical and biosensing applications. Previously, a nitrite sensor was developed using Au NPs/poly(dimethyl diallyl ammonium chloride)-modified titanium carbide MXene (Ti_3_C_2_T-PDDA). The AuNPs/Ti_3_C_2_T-PDDA composite exhibited synergistic effects [124]. The high catalytic activity of AuNPs, the large surface area and excellent conductivity of Ti_3_C_2_T_x_, and the electrostatic interactions from PDDA improved the sensing of nitrite. Linear ranges of 0.1 to 2490 μM and 2490 to 13,490 μM with an LOD of 0.059 μM were achieved for nitrite detection. In another study [125], a selective and sensitive nitrite electrochemical sensor was also developed by using Pd-Cu nanospheres/molybdenum carbide as sensing materials. The Pd-Cu-Mo_2_C/GCE demonstrated a linear range of 5 to 165 nM, an LOD of 0.35 nM, and sensitivity of 3.308 μA·nM^−1^·cm^−2^ for nitrite detection. Furthermore, this electrode was also employed for the monitoring of nitrite in real samples (sausages, river water, and milk) which showed satisfactory recoveries. Chen et al. [126] also prepared stearyl trimethyl ammonium bromide-functionalized niobium carbide@MWCNTs (Nb_2_C@MWCNTs-STAB) for sensing applications. The Nb_2_C@MWCNTs-STAB-modified electrode demonstrated two wide linear ranges of 0.1 to 100 μM and 100 to 2000 μM with an LOD of 0.022 μM. The real sample tests in milk and spinach yielded 89.8 to 104.5% recovery. The xylan-derived carbon quantum dots (CQDs)-capped Au NPs were prepared and combined with MXene to form Au@CQDs-MXene for nitrite detection, which exhibited a decent electrochemical performance [127]. This electrode can be used for the determination of nitrite in food samples. The hierarchical NiFe-LDH/Mo_2_C (LDH = layered double hydroxide) composite was synthesized through the green solvothermal method [128]. The fabricated sensor demonstrated a broad linear range of 0.005 to 260 μM, an LOD of 0.0017 μM, and sensitivity of 3.6 µA µM^−1^ cm^−2^. The synergistic interactions of the fabricated electrode displayed an improved sensing performance for nitrite detection. Habibi et al. [129] prepared carbon ceramic electrodes (CCEs)-modified MWCNTs-Co/Zn ZIFs and MXene-Co/Zn ZIFs electrodes for the determination of nitrite. It was observed that both electrodes exhibited significant electro-catalytic activity, but MXene-Co/Zn ZIFs/CCE outperformed MWCNTs-Co/Zn ZIFs/CCEs due to the synergistic effect between the 2D MXene and Co/Zn-ZIF. The MXene-based sensor demonstrated a linear range of 2 to 500 μM and an LOD of 1.6 μM with sensitivity of 1.80180 μA mM^−1^ cm^−2^. This indicates that MXene-based materials can be explored for the construction of nitrite electrochemical sensors. The sensing performance of the MXenes and composites-based sensors is presented in Table 6.

## 3. Conclusions and Future Trends

In summary, it can be stated that significant progress has been made in developing advanced electrode materials for electrochemical nitrite-sensing applications. Various electrode modifiers such as metal oxides (ZnO, CeO_2_, Co_3_O_4_, Fe_2_O_3_, CuO, and TiO_2_), metal sulfides (MoS_2_), carbon-based materials (CNTs, rGO, and g-CN), polymers, MOF/ZIF-derived composites, and MXenes have been used for nitrite-sensing studies. The above-mentioned studies on nitrite detection exhibited excellent sensitivity, selectivity, stability, and low detection limits. These electrode materials have enabled the fabrication of cost-effective, portable, and reliable sensing platforms suitable for environmental and food-safety monitoring. The enhanced electrochemical performance of these electrodes is mainly attributed to their large surface area, active sites, tunable electronic properties, and synergistic interactions between hybrid components, which may facilitate electron transfer and improve the oxidation of nitrite. Moreover, the integration of nanostructured composites and heterojunctions has improved the kinetics and improved anti-interference properties. Despite these achievements, several challenges, including the low conductivity of metal oxides, poor stability of polymers or MOFs in acidic conditions, and the use of harsh conditions for MXene synthesis, hinder the real-world applicability of the reported nitrite sensors. Other challenges also include selectivity in similar interfering species, long-term stability, reproducibility, scalability, high cost, and environmental sustainability. We believe that future research may overcome such issues and may achieve the successful integration of electrochemical sensors with wearable and portable smart devices. Moreover, smartphone-controlled electrochemical sensing devices could be developed for environmental monitoring and food safety applications. Machine learning technology should be integrated with electrochemical sensing techniques to optimize and enhance the performance of nitrite sensors.

## Figures and Tables

**Table 1:** Electrochemical parameters of the ZnO-based sensors for nitrite detection.

Electrode Material	LOD (µM)	Linear Range (µM)	Sensitivity	Sensing Method	References
Ag-Cu@ZnO	17	0 to 1500	-	LSV	[41]
rGO/ZnO/GCE	1.18	200 to 4000	0.3156 μA μM^−1^ cm^−2^	LSV	[42]
rGO/ZnO/GCE	1.36	20 to 520	0.2754 μA μM^−1^ cm^−2^	Amperometry	[42]
ZnO/Nafion/GCE	0.62	0.8 to 4860	0.392 μA μM^−1^ cm^−2^	LSV	[43]
ZnO/Nafion/GCE	0.21	0.3 to 6140	0.524 μA μM^−1^ cm^−2^	Amperometry	[43]
Sb-ZnO/N-rGO/GCE	0.43	0.4 to 900 and 1000 to 5000	397.6 μA mM^−1^ cm^−2^	LSV	[44]
Sb-ZnO/N-rGO/GCE	0.13	0.2 to 1200 and 1500 to 7800	294.4 μA mM^−1^ cm^−2^	Chronoamperometry	[44]
Near-spherical ZnO/GCE	0.89	1.9 to 800 and 1080 to 5900	0.646 μA μM^−1^ cm^−2^	LSV	[45]
Near-spherical ZnO/GCE	0.39	0.6 to 220 and 460 to 5500	0.785 μA μM^−1^ cm^−2^	Chronoamperometry	[45]
PPy/TiO_2_/ZnO/GCE	0.14	1 to 20	70.3234 μA μM^−1^ cm^−2^	LSV	[46]
PA-TaCoPc@ZnO	0.021	1 to 10	6.3575 μA μM^−1^ cm^−2^	Chronoamperometry	[47]
ns-ZnO/N-rGO/GCE	0.29	0.1 to 4600	405.8 μA mM^−1^ cm^−2^	LSV	[48]
ns-ZnO/N-rGO/GCE	0.08	0.037 to 5900	301.9 μA mM^−1^ cm^−2^	Chronoamperometry	[48]
Ms−Au/ZnO@Pt−CC	0.09	0.2 to 4986	5677 μA mM^−1^ cm^−2^	Amperometry	[49]
cl-ZnO-nafion/GCE	0.32	0.00095 to 0.515 and 0.667 to 5.41	1336.1 µA mM^−1^ cm^−2^	LSV	[50]
cl-ZnO-nafion/GCE	0.26	0.0008 to 0.462 and 0.608 to 7.84	824.6 µA mM^−1^ cm^−2^	Chronoamperometry	[50]

**Table 2:** Electrochemical parameters of the Co_3_O_4_- and CeO_2_- and iron oxide-based sensors for nitrite detection.

Electrode Material	LOD (µM)	Linear Range (µM)	Sensitivity	Sensing Method	References
ZnO-nf-nafion/GCE	0.28	0.0008 to 4.81	345.5 µA mM^−1^ cm^−2^	LSV	[51]
ZnO-nf-nafion/GCE	0.11	0.0002 to 0.453 and 0.57 to 8.5	763.4 µA mM^−1^ cm^−2^	Chronoamperometry	[51]
L-Arginine/Co_3_O_4_/FTO	0.00195	10 to 16,000	158 μA mM^−1^	Amperometry	[52]
MoO_3_/Co_3_O_4_/CC	0.075	-	1704.1 μA mM^−1^ cm^−2^	Amperometry	[53]
Sn-CeO_2_	0.016	10 to 6000	245.4 and 89.53 µA mM^−1^	Chronoamperometry	[55]
CB/NiCo_2_S_4_@CeO_2_/GCE	0.003	0.2 to 7400	470 µA mM^−1^ cm^−2^	Amperometry	[56]
Cu@CeO_2_−rGO	0.0101	10 to 2000	1963.2 µA mM^−1^ cm^−2^	Amperometry	[57]
CeO_2_ NSs/CuO NFs/CC	0.0347	0.1 to 4000	11,610 µA mM^−1^ cm^−2^	Amperometry	[58]
Fe_3_O_4_@Au@Cys/rGO/GCE	0.008	0.03 to 2215	-	DPV	[59]
NiO/Fe_2_O_3_/GCE	0.05	5 to 500	-	DPV/Amperometry	[60]
CSs@Fe_2_O_3_/Fe_3_C/Fe	0.06	1 to 2540	451.85 µA mM^−1^ cm^−2^	Amperometry	[61]
Fe_3_O_4_@SiO_2_(acid-treated)/GCE	3.33	10 to 1000	-	DPV	[62]
Fe_3_O_4_@Au/MOF-(P_2_W_17_V)_6_/GCE	0.532	0.01 to 100	11.682 µA µM^−1^ cm^−2^	CV	[63]
GO@Fe_2_O_3_/Y_2_O_3_ NCs/Nafion/GCE	2250	0.74 M to 1.09 M	73.83966 µA mM^−1^ cm^−2^	LSV	[64]

**Table 3:** Electrochemical parameters of the CuO-, TiO_2_-, and MoS_2_-based sensors for nitrite detection.

Electrode Material	LOD (µM)	Linear Range (µM)	Sensitivity	Sensing Method	References
CuO/NiO/FTO	0.013	1 to 1800	7.2 mA mM^−1^ cm^−2^	Amperometry	[65]
CuO NPs/CC	0.043	0.5 to 3000	1656 μA mM^−1^ cm^−2^	Amperometry	[66]
CuO@NF-1	28.7	1 to 4250	2.402 mA mM^−1^ cm^−2^	Chronoamperometry	[67]
Ti_3_C_2_T_X_/CTAB/CS/GCE	0.85	3 to 250 and 250 to 1250	-	DPV	[68]
1.0% [MTZ_2_] GPS	0.00012	0.0004 to 0.01	-	SWV	[69]
Au-doped TiO_2_ NPs	0.095	3.3 to 120	-	SWV	[70]
Ni/MoS_2_/GCE	2.74	20 to 1000	0.01509 μA μM^−1^	DPV	[72]
Oxidized MoS_2_ Nanosheets	0.028	1 to 386	-	Chronoamperometry	[73]
Au_4.5_NPs@MoS_2_ /rGO/GCE	0.804	0.2 to 2600 and 2600 to 16,000	0.805 and 0.468 μA μM^−1^ cm^−2^	Amperometry	[75]
GO/PEDOT:PSS/GCE	0.5	1 to 200	-	DPV	[76]
3D MoS_2_/2D C_3_N_4_/GCE	0.065	0.1 to 1100	-	DPV	[77]

**Table 4:** Electrochemical parameters of the polymer-, MOF-, and ZIF-based sensors for nitrite detection.

Electrode Material	LOD (µM)	Linear Range (µM)	Sensitivity	Sensing Method	References
Au-PPy-C/g-C_3_N_4_ NCs/GCE	1.11	1.5 to 22.5	91.19 μA μM^−1^ cm^−2^	DPV	[79]
AuNRs/ErGO/PEDOT:PSS/GCE	0.2	0.8 to 100	0.0451 μA μM^−1^	DPV	[80]
AuNRs/ErGO/PEDOT:PSS/GCE	0.08	0.2 to 100	0.0634 μA μM^−1^	DPV	[80]
PEDOT-C@Cu-NPs	3.91	5 to 580	0.6372 μA μM^−1^ cm^−2^	Amperometry	[81]
AuNPs@PPy/rGO/GCE	0.0165	0.005 to 82	-	DPV	[83]
Ni/Co,N-CP/GCE	0.094	1 to 500	-	Chronoamperometry	[84]
CoN-PCRs-0.6	0.14	0.2 to 4000 and 4000 to 10,000	-	Amperometry	[86]
ZIF-67C@RGO/NiNPs/GCE	0.086	0.2 to 123 and 123 to 473	-	Amperometry	[87]
CoN-CRs/MGCE	0.17	0.5 to 4000 and 4000 to 8000	1.03 and 0.82 μA μM^−1^ cm^−2^	Amperometry	[88]
CB/Cu-MOF/SPCE	0.084	1 to 200	-	LSV	[90]
C-A Zn/Co-Fe PNSs@CC	0.44	-	-	Amperometry	[91]
ZrCu-MOF-818/ILs	0.148	6 to 5030	-	DPV	[92]
Cu-MOF	16.39	0.05 to 1000	2.91 μA μM^−1^ cm^−2^	Chronoamperometry	[93]
ZIF-8	24.48	0.05 to 1000	1.56 μA μM^−1^ cm^−2^	Chronoamperometry	[93]
Ag-MOF/GCE	0.045	4 to 4040	-	SWV	[94]
Ag-MOF/GCE	23	5 to 5900	-	CV	[94]
Ni-PDCA/SPCE	0.052	0.1 to 1000	240 µA mM^−1^ cm^−2^	DPV/Amperometry	[96]
H-CoMnN-PCs	0.097	0.1 to 1500 and 1500 to 12,000	1.2644 and 0.9073 μA μM^−1^ cm^−2^	Amperometry	[97]

**Table 5:** Electrochemical parameters of the gCN-, CNTs-, GO-, and rGO-based sensors for nitrite detection.

Electrode Material	LOD (µM)	Linear Range (µM)	Sensitivity	Sensing Method	References
Chitosan/g-C_3_N_4_/GCE	0.021	40 to 2000	-	DPV	[98]
Chitosan/g-C_3_N_4_/GCE	0.010	20 to 4230	-	Amperometry	[98]
GCE/(EDAS/g-C_3_N_4_-Au)_NCM_	0.6	10 to 375	0.0696 µA µM^−1^ cm^−2^	Amperometry	[100]
LIG/f-MWCNT-AuNPs	0.9	10 to 140	-	SWV	[101]
1,1′-(1,4-Butanediyl)dipyridinium (bdpy)PW_11_Co/MWCNTs-COOH/GCE	0.63	10 to 1600	17.9 µA mM^−1^	Amperometry	[102]
GCE/MWCNTs/AuNPs/PM. MWCNTs	0.041	0.4 to 1475	-	DPV/Amperometry	[104]
*MEC*/CG-MWCNT-Pd−2E-Peaemp	0.39	2.48 to 909.12	-	DPV	[105]
Y/Fe-MWCNT/GCE	0.027 M	1 to 1.66 M	6.205 µA mM^−1^ cm^−2^	LSV	[106]
CuPc-N-MWCNTs-SPCE	10	50 to 1000	-	DPV	[107]
AuNPs/MWCNT-OH/graphene/GCE	3.64	40 to 1000	-	DPV	[108]
ERHG/GCE	0.054	0.2 to 10,000	0.311 µA µM^−1^ cm^−2^	Amperometry	[109]
GO-PANI-AuNPs/GCE	0.17	0.0005 to 0.24 and 0.24 to 2.58		Amperometry	[110]
Cu^2+^/NP-LIG	0.9	2 to 1000	-	DPV	[111]
hematene/GO	2	2 to 1000	-	CV/i-t	[114]
LSM/rGO	0.016	2 to 100 and 100 to 5000	0.041 and 0.039 µA µM^−1^ cm^−2^	Amperometry	[115]
Au/NiO/Rh/LIG	0.3	1 to 1000	2292.99 µA mM^−1^ cm^−2^	LSV	[116]
Au/NiO/LG	0.98	2 to 850	2104.64 µA mM^−1^ cm^−2^	LSV	[116]
Au/Rh/LIG	1.06	3 to 800	1850.32 µA mM^−1^ cm^−2^	LSV	[116]
rGO/CuS	0.0022	5 to 8000	2002 µA mM^−1^ cm^−2^	Amperometry	[119]
B-PG	1.1	3 to 15,000	-	Amperometry	[122]
CuCo_2_O_4_/rGO	0.00082	10 to 4500 and 4500 to 10,300	-	Amperometry	[123]

**Table 6:** Electrochemical parameters of the MXene-based sensors for nitrite detection.

Electrode Material	LOD (µM)	Linear Range (µM)	Sensitivity	Sensing Method	References
AuNPs/Ti_3_C_2_T_x_-PDDA	0.059	0.1 to 2490 and 2490 to 13,500	-	Amperometry	[124]
Pd-Cu-Mo_2_C/GCE	0.00035	0.005 to 0.165	-		[125]
Nb_2_C@MWCNTs-STAB	0.022	0.1 to 100 and 100 to 2000	-	DPV	[126]
Au@CQDs-MXene/GC	0.078	1 to 500 and 500 to 3200	-	DPV	[127]
NiFe-LDH/Mo_2_C	0.0017	0.005 to 49 and 49 to 260	3.60 µA µM^−1^ cm^−2^	Amperometry	[128]
MXene-Co/Zn ZIFs/CCE	1.6	2 to 500	1.80180 µA mM^−1^ cm^−2^	Amperometry	[129]
